# Supplementary material for: The interactome of intact mitochondria by cross-linking mass spectrometry provides evidence for coexisting respiratory supercomplexes
Source: Mol Cell Proteomics. 2017 Dec 8;17(2):216–32. doi: 10.1074/mcp.RA117.000470 (PMC5795388; doi:10.1074/mcp.RA117.000470)
Supplement: Supplemental Data [file supp_RA117.000470_133922_0_supp_23978_fzffwf.zip › spectra_annotation/mito_DR_spectra_annotation/432-1-19-1-1-1.pdf]

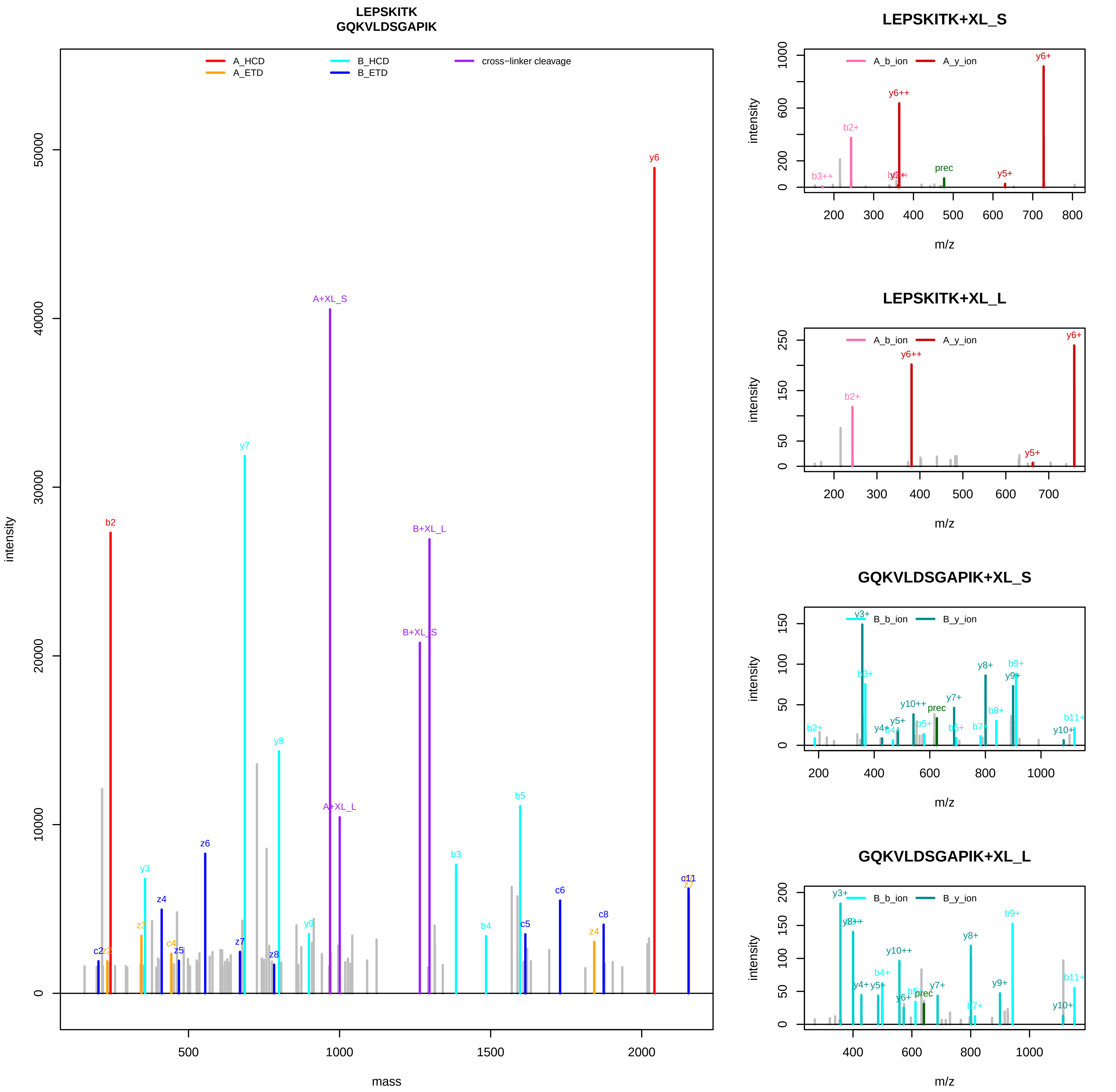
LEPSKITK
GQKVLDSGAPIK
A_HCD
A_ETD
B_HCD
B_ETD
cross−linker cleavage
intensity
mass
y6
A+XL_S
y7
B+XL_L
b2
B+XL_S
y8
A+XL_L
b5
b3
z6
y3
z4
c6
c11
z7
c8
c5
b4
z3
y9
z4
c4
z7
z5
c2
z2
z8
LEPSKITK+XL_S
A_b_ion
A_y_ion
m/z
LEPSKITK+XL_L
GQKVLDSGAPIK+XL_S
B_b_ion
B_y_ion
GQKVLDSGAPIK+XL_L
prec
